# Longevity factors for short and extra-short implants: An eight-year retrospective observational clinical study

**DOI:** 10.4317/medoral.27712

**Published:** 2025-11-22

**Authors:** Sérgio José Tavares Leite, Marcelo Harduin, Marcelo Rizzato, Alexandre Oliveira Gonçalves, Juliana Campos Hasse Fernandes, Gustavo Vicentis Oliveira Fernandes

**Affiliations:** 1 Friburguense Institute of Postgraduation - Rio de Janeiro, Brazil; 2 Private Researcher, St. Louis, MO 63104, USA; 3 Still University - Missouri School of Dentistry &amp; Oral Health, St. Louis, MO, USA

## Abstract

**Background:**

This study aimed to evaluate implants placed in the mandible and maxilla and analyze the correlation between local and systemic factors that affect the clinical and prosthetic performance of short and extra-short implants.

**Material and Methods:**

Implants were analyzed based on 18 factors: Location (anterior or posterior; maxilla or mandible); presence or not of previous grafting; bone quality; prosthesis was/was not installed immediately; type of prosthetic connection (external hexagon or Morse Taper); thread type (trapezoidal, triangular, or hybrid); surface's characteristic; implant length; implant width; prosthesis installation follow-up; type of prosthesis retention (cemented or screw-retained); single prosthesis or splinted to another implant; antagonist occlusion type; presence or absence of intermediary prosthetic component; prosthetic abutment height; distance between intermediaries component; presence or absence of implant bicortilization; and implant insertion torque. Clinical intraoral analysis included dimensions of the occlusal part and the inclination of the cusps (15 degrees versus &gt;15 degrees). Possible systemic influences were also evaluated. Patient satisfaction was assessed through a questionnaire. The statistical analysis considered results significant if p&lt;0.05.

**Results:**

This study analyzed 91 dental implants (60 short/extra-short and 31 standard/long) placed in 16 patients, including individuals with diabetes (n=3), smoking (n=1), and parafunctional habits (n=7). Implants were distributed across the maxilla (43.3%) and mandible (56.7%) arches, with one short implant failure (survival rate = 98.3%). The mean peri-implant bone loss was 6.80±13.06mm² for short/extra-short and 8.19±12.10mm² for standard/long implants. Bone loss was lower in males (3.32±6.03mm²) than in females (7.61±8.55mm²), and implant diameter influenced the osseointe-gration loss area, highlighting relevant biomechanical and risk-related factors. Significantly reduced peri-implant bone loss was observed in implants with abutments &gt;2mm, Morse taper connections, bicorticalization, insertion torque 35N, anterior placement, maxillary location, and prior bone grafting (p&lt;0.05 for all). These findings suggest that such variables may positively influence os-seointegration and support the long-term success of short implants.

**Conclusions:**

The use of short- and extra-short implants is a feasible treatment option for mid- and long-term rehabilitation of the full and partial maxillary and mandibular arches.

## Introduction

Dental implants have revolutionized contemporary dentistry. With the understanding of the osseointegration process and its evaluation (1), significant advancements have been made in both the science and technology of implantology. The predictability and effectiveness of dental implants in the partial or full rehabilitation of edentulous patients have been extensively studied and confirmed in the literature (2); as a result, the indications for implant therapy have expanded considerably (3). The success rate of implants has increased from approximately 85% in the 1980s to over 95% today, regardless of implant site (2 , 3).

However, the dramatic increase in the number of implants placed over the past three decades has raised several clinical challenges. One critical issue is how to maintain the long-term health, function, and aesthetics of short and extra-short implants, which are increasingly used in anatomically compromised sites (3 , 4). It is important to recognize that while implants replicate the function of natural teeth, their structural and biological interfaces differ significantly. Unlike natural teeth, im-plants lack periodontal ligaments and cementum, exhibit reduced vascularity, and present a sub-gingival emergence profile with connective tissue fibers running parallel to the implant surface. These factors render implants more susceptible to inflammation and bone loss when exposed to plaque accumulation or microbial invasion (5).

As a result of these anatomical and histological differences, meticulous maintenance is required to preserve peri-implant health. Neglecting these requirements can lead to the loss of osseointegration and failure of both hard and soft tissue support (6). The long-term success of short and extra-short implants is highly dependent on the quality of osseointegration and the appropriate application of occlusal loading (3); early detection of marginal bone loss (MBL) is therefore essential to prevent implant failure.

MBL around implants can be categorized as early or late. Early bone loss typically occurs during the healing phase or within the first year after prosthetic loading and may compromise initial osseointegration. Contributing factors include bone quality, surgical trauma, occlusal overload, microgaps at the implant-abutment interface, and violation of the biological width. Late bone loss, on the other hand, is characterized by the gradual loss of marginal bone following successful osseointegration and may threaten the long-term stability of the implant. Although peri-implantitis and occlusal overload are considered the most likely etiologies of late bone loss (7), the literature remains in-conclusive regarding this relationship.

Rehabilitation of the posterior maxilla and mandible with implants can be particularly challenging due to reduced bone volume resulting from resorption after tooth extraction or anatomical limitations (3). In such cases, the placement of standard-length implants may be contraindicated due to proximity to critical anatomical structures, such as the maxillary sinus or the inferior alveolar nerve.

Sinus floor elevation procedures, using either lateral or transcrestal approaches, have been widely validated as effective techniques for increasing available bone height prior to implant placement in the posterior maxilla (8). Despite high reported survival rates, these procedures are associated with a relatively high risk of complications, including sinus membrane perforation (20-44% in the lateral window approach), which may be underreported in the transcrestal approach due to its blind/closed technique. Other complications, although less frequent, include postoperative infection and bone graft failure. A thorough understanding of maxillary sinus anatomy and careful preoperative eval-uation are essential to minimizing such risks (9).

The use of short implants has emerged as a promising alternative to bone augmentation proce-dures, particularly in the posterior regions of the jaws. Short implants are generally defined as those less than 10mm in length, while extra-short implants measure less than 7mm (10). Although earlier studies suggested unpredictable outcomes with short implants (6), more recent systematic reviews and clinical trials have reported favorable long-term success rates, especially when proper case selection and loading protocols are followed. Despite a higher incidence of prosthetic complications, short and extra-short implants placed in the posterior jaws have demonstrated high survival rates and may reduce patient morbidity associated with advanced grafting procedures (11).

Thus, this retrospective observational clinical study aimed to evaluate the implants placed in the mandible and maxilla and analyze the correlation between local and systemic factors affecting the clinical and prosthetic performance of short and extra-short implants. The null hypothesis (H0) was that there was no difference in mean bone loss between the groups; the positive hypothesis was that short and extra-short implants achieved a significant difference in mean bone loss compared to the other groups.

## Material and Methods

This retrospective observational clinical study was designed and conducted in accordance with the principles of the Declaration of Helsinki (1975, updated 2013), as revised by the World Medical Association for biomedical research involving human beings in 2000. Ethical approval of the study was obtained by the research ethics committee of the Faculdade São Leopoldo Mandic (Campinas, São Paulo, Brazil; 2.268.057). All patients were informed about the objectives and study design and signed an informed consent form.

Eligibility criteria

The criteria were evaluated based on the medical records of all patients who underwent implant placement (short and extra-short implants) and prosthetic rehabilitation during the Course in Implantology at the Friburguense Institute of Postgraduate Medical and Dental Sciences (IFPG; Nova Friburgo, Rio de Janeiro, Brazil). The patient inclusion criteria were: Undergone the rehabilitation (implant and prosthetic procedures) at the IFPG; the presence of implants with a length 9mm; the presence of periapical radiography at the time of installation of the prosthetic crown; over 18 years old; ability to sign the informed consent form; and rehabilitation (installation of the crown in occlusion) in 2017.

Patients were excluded if they had one of the following criteria: The absence of implants with the desired length (short or extra-short implants); the presence of the prosthetic crown installed less than a year; the absence of periapical radiography at the time of installation of the prosthetic crown; inability/refuse to sign the informed consent form; inability or unwillingness to return for follow-up visits.

Variables in analysis: Clinical and radiological assessment

Implants were analyzed based on 18 factors: Location (anterior or posterior; maxilla or mandible); presence or not of previous grafting (maxillary sinus lift; vertical bone augmentation); bone quality (bone density types I, II, III, or IV); whether the prosthesis was/was not installed immediately after the implant placement; type of prosthetic connection (external hexagon or morse taper); thread type (trapezoidal, triangular, or hybrid); surface's characteristic (smooth or rough); implant length (short [7mm and 9mm] or extra-short [4mm and 6mm]) (19); implant width (narrow [&lt;3.75mm], regular [3.75 to 4.1mm] or wide [&gt;4.1mm]); prosthesis installation follow-up; type of prosthesis retention (cemented or screw-retained); single prosthesis or splinted to another implant; antagonist occlusion type; presence or absence of intermediary prosthetic component; prosthetic abutment height (considering the platform of the implant as reference); distance between intermediaries component; presence or absence of implant bicortilization (achieving anchorage at the maxillary sinus floor or the nasal cavity floor); and implant insertion torque (a new and calibrated wrench torque provided by the same company of the implants).

Clinical intraoral analysis was performed, including dimensions of the occlusal part (buccolingual and mesiodistal distances), and the inclination of the cusps (15 degrees versus &gt;15 degrees); pan-oramic radiographs were taken to analyze the crown/implant relationship (measurements and proportion). Possible systemic influences were evaluated, including the age of patients (&gt; or &lt; 60 years), presence of parafunctional habits, smoking, diabetes, and accumulation of bacterial plaques. The effects of these agents, especially regarding peri-implant bone loss, were assessed by a blinded, external, and experienced examiner.

**Figure 1:**
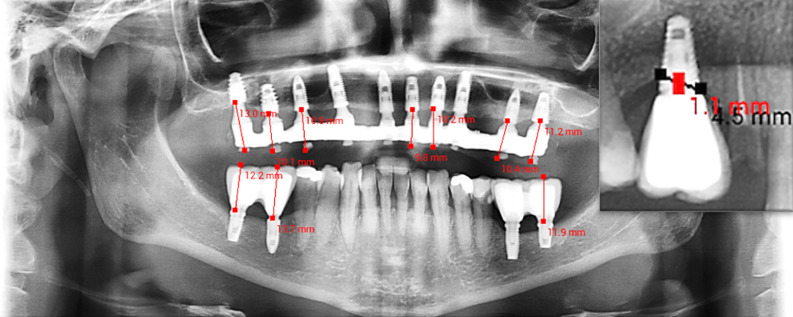
Panoramic radiograph with measurements for the height of the prosthetic rehabilitation of short and extra-short implants, and measurement of the peri-implant bone loss.

The clinical evaluation was carried out at least one year after the installation of the definitive prosthesis. Survival and success rates were evaluated and recorded, following the definitions and parameters described and already established in the literature (2). Any prosthetic or clinical com-plication was recorded. Prosthetic success was evaluated based on the following criteria: The prosthesis was in function, without mobility issues, and there was no pain. The radiographic evaluation was performed using digital panoramic radiographs taken on a computed tomography (CT) scanner to analyze the crown-to-implant ratio (measurements and proportion) (Figure 1), as well as to measure MBL around implants over time. Measurements were performed with the Radi-oLaudo+ system (Radio Memory, Belo Horizonte, Minas Gerais, Brazil).

1

All radiographs were acquired using the same digital panoramic unit, in accordance with a stand-ardized patient positioning protocol that included bite-block stabilization and head alignment guides to minimize distortion. To account for equipment-specific magnification, all images were calibrated using the known implant length provided by the manufacturer, which was measured directly from the radiograph. This allowed for the correction of the magnification factor before linear measurements were recorded.

Marginal bone levels were measured from the implant platform (reference line) to the most coronal point of bone-to-implant contact on the mesial and distal aspects. When bone resorption was present, the vertical distance was recorded as MBL. From this point, a measurement was made at the center of the implant toward the implant platform (Figure 1). Maintaining osseointegration across the entire implant surface is essential for successful rehabilitation. Therefore, we will use the terms "loss of osseointegration" or "peri-implant bone loss" to refer to the absence of bone structure that should be contiguous with the treated implant surface.

To verify the reproducibility of the radiograph assessment, 25% of the sample radiographs were re-analyzed. One examiner repeated the measurements after two weeks (intra-observer reliability), and another examiner independently assessed the same radiographs (inter-observer reliability). Both examiners were responsible for analyzing the radiographs throughout the study. Intra- and inter-observer intraclass correlation coefficients (ICC, two-way mixed, absolute agreement) were excellent: ICC =0.94 (95% CI: 0.89-0.97) for intra-observer and ICC =0.91 (95% CI: 0.85-0.95) for inter-observer agreement. Bland-Altman analysis showed minimal bias (-0.02mm intra-observer, +0.03mm inter-observer) and narrow limits of agreement, confirming high reproducibility of the radiographic MBL measurements.

**Figure 2:**
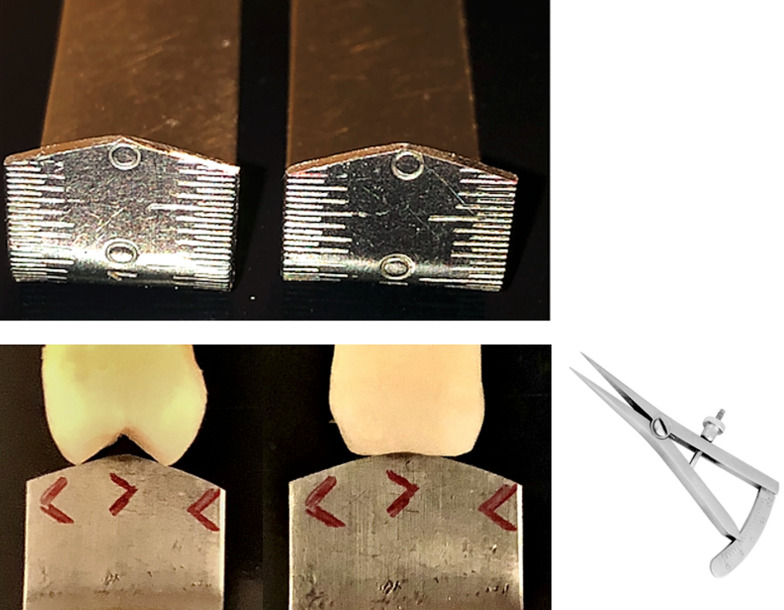
Modified endodontic ruler; cusp angulation (left: &gt; 15°; right: ≤ 15°); thickness gauge.

An initial clinical evaluation was performed to assess oral hygiene and the quality of the gingival tissue around the implant-supported dentures. The angulation of the denture cusps was evaluated using a modified endodontic ruler to achieve a 15° angulation (Figure 2). The modified ruler was placed on the occlusal surface of the molar and premolar dentures. If the tip of the modified ruler did not touch the bottom of the mesiodistal sulcus, but only the grinding slopes of the cusps, we had an angulation greater than 15° (Figure 2). When the tip of the modified ruler touched the bottom of the sulcus, rather than the grinding slopes of the cusps, the angulation was 15° or less (Figure 2). When the tip and sides of the modified ruler touched the bottom of the sulcus and grinding slopes sim-ultaneously, we had an angulation of 15°.

2

A thickness gauge (Figure 2) was used to assess the mesiodistal and buccolingual distances of the prosthetic crown. The buccolingual distance was measured through the prosthetic equator of the crown, and the mesiodistal distance was measured between the mesial and distal marginal ridges. The linear measure of osseointegration loss was obtained.

Patient satisfaction

A questionnaire on satisfaction with the dental implant and rehabilitation experience was distributed to all participants. The questionnaire consisted of one question for each of the following six categories: Comfort, appearance, ability to chew food, ability to speak, ability to clean the crown on the implants, and overall satisfaction. For each question, there were four possible scores: Excellent, good, fair, and poor. If the patient had multiple implants, it was requested to provide the worst experience.

Statistical analysis

Statistical analyses were conducted using R (version 3.6, 2019) and SPSS (version 20.0, SPSS Inc., Chicago, IL, USA). Continuous data were expressed as mean ± standard deviation (SD). For two-group comparisons, independent t-tests or Mann-Whitney U tests were used depending on data normality and variance assumptions. A significance level of p&lt;0.05 was adopted.

To account for the non-independence of multiple implants within the same patient, a multilevel mixed-effects model (random-intercept model) was employed. In this model, implants were treated as level-1 units, nested within patients (level-2 units), allowing the model to control for intra-patient variability. Patient ID was included as a random effect to adjust for correlated outcomes among implants from the same patient.

This approach accounts for intra-class correlation (ICC) due to clustering and prevents underestimation of standard errors that may arise from ignoring such correlations. Mixed-effects linear regression was used for continuous outcomes (e.g., MBL), and generalized linear mixed models with appropriate link functions were considered for binary outcomes (e.g., implant success/failure). Effect sizes were also estimated using Cohen's d, categorized as small (0.2), moderate (0.5), large (0.8), and very large (greater than 1.2). The mixed-effects models allow robust comparisons while adjusting for both fixed effects (e.g., implant type, abutment height, arch, connection type) and random effects (patient-level variability).

Marginal bone loss (MBL, mm) was analyzed using a linear mixed-effects model with a random intercept for patients to account for clustering of implants within patients. Fixed effects were pre-specified based on clinical relevance. They included implant length category (short/extra-short vs standard/long), abutment height (&lt;2mm vs 2mm), prosthetic connection (Morse taper vs external hexagon), implant diameter (continuous), arch (maxilla vs mandible), region (anterior vs posterior), antagonist type, bone grafting, insertion torque, loading protocol (immediate vs conventional), follow-up time (months, continuous), age, sex, smoking, diabetes and parafunction. Continuous predictors were centered. We prioritized the a priori model (all clinically relevant covariates retained); sensitivity analyses included a reduced model (covariates with p&lt;0.20 in univariable screening) and an exploratory penalized selection (LASSO). Collinearity was assessed using variance inflation factors (VIF) from an analogous OLS model; predictors with VIF values greater than 5 were in-spected and handled based on clinical rationale. Random-effect variance components were estimated via restricted maximum likelihood (REML), and ICC was calculated as patient variance / (patient variance + residual variance). Model diagnostics included residual normality (QQ plots), heteroscedasticity checks, and influence diagnostics. All analyses were performed in R (packages lme4, lmerTest, broom.mixed, performance, car).

Variance Inflation Factor (VIF) estimation was performed. The VIF quantifies the extent to which the variance of a regression coefficient is inflated due to multicollinearity. A VIF of 1 indicates no col-linearity; a VIF between 1 and 5 suggests moderate correlation, often considered acceptable; a VIF &gt; 5 indicates high collinearity, which can destabilize a regression model.

## Results

A total of 91 implants were included in the study, with 16 patients enrolled (4 males and 12 females), aged between 37 and 75 years. The median follow-up was 19 months (interquartile range, IQR, 16.0-21.0; range of 12-24 months). Of these, three patients were diabetic, one was a smoker, and seven had parafunctional habits (bruxism or clenching). Sixty were short and extra-short implants, and 31 were standard and long implants. Twenty-six implants were placed in the maxilla (43.3%): 15 implants in the posterior region (premolars and molars) and 11 implants in the anterior region (incisors and canines); 34 were mandibular (56.7%): 27 implants were placed in the posterior region and seven in the anterior region. Only one short implant failed, resulting in a 98.3% survival rate (95% CI: 93.8%-99.8%); the failed implant did not belong to the high-risk group (smokers, diabetics, parafunctional habits, or excessive plaque accumulation); it was placed in type I bone and was part of a splinted prosthesis on two elements. This implant was initially considered lost during the evaluation for this study, after 24 months; however, its data were validated to obtain the most accurate total after 24 months. It was subsequently removed, and the patient was rehabilitated. Of the sixty short and extra-short dental implants, the diameters ranged from 3.5mm (narrowest) to 5.0mm (widest); lengths ranged from 5 to 9mm; 20 external hexagon implants and 40 Morse taper implants; 19 (31.7%) showed peri-implant bone loss. Additionally, 31 standard and long implants had lengths ranging from 10mm to 15mm. The sample of short and extra-short implants included. The average marginal bone loss (MBL) was 0.51±0.98mm for short and extra-short implants, and 0.61±0.90mm for standard and long implants, with an overall mean MBL of 0.55±0.95mm for the 91 implants included in this study (Table 1). In males, the mean MBL was 0.25±0.46mm, compared to 0.57±0.64mm in females. Considering only 1mm of vertical bone loss, implants with diameters of 3.5mm and 5.0mm would correspond to theoretical circumferential losses of approximately 1.0mm and 0.8mm, respectively. These data are relevant for both assessing longevity and identifying risk factors associated with it.Table 1

The short and extra-short-implant group demonstrated better performance, with a smaller average area of osseointegration loss. However, longer implants had a proportionally higher remaining area of osseointegration. The 60 short and extra-short implants had their prostheses retained by screws, none of which were cemented in place. All had prosthetic abutments installed, and none had a smooth surface. Only two implants placed were extra-short, and only one received immediate prosthetic loading; scarce data were available for these two factors. Then, the report of their out-comes consisted only of exploratory observations. Because the subgroup size (extra-short, n=2) is too small for meaningful statistical inference or generalization, no categorical claims about the performance or safety of extra-short implants are made. These cases are described for completeness and hypothesis-generation; larger, adequately powered studies are required to evaluate extra-short implants definitively. Other criteria evaluated yielded similar comparative results, providing no relevant evidence for the study. These were: Four systemic factors (parafunctional habits, plaque accumulation, diabetes, and smoking); bone quality; cusp inclination &gt; or 15°; occlusal table dimension; thread types (triangular, trapezoidal, or hybrid); the proportion between the prosthetic crown and the implant; the distance between the abutments, prosthetic function time, and variation in implant diameter. On the other hand, 10 out of 18 factors presented relevant comparative values. The short and extra-short implants showed a lower average MBL when evaluated by each factor. The four implants that occluded with a complete denture did not exhibit bone loss; the other four, which did not have an antagonist, presented with an average MBL of 0.38mm. Twenty implants occluding with other implants presented 0.31mm, whereas 32 occluding with teeth showed 0.72mm (Table 1). The factors listed in Table 1 reflected specific circumstances, with possible exceptions being the choice of prosthetic abutment height and the type of prosthetic connection, which are often planning decisions made by the dentist. These choices appeared to affect implant longevity, particularly for short and extra-short implants, as the data collected suggests. The 60 prosthetic abutments of the short and extra-short implants, as well as the 31 of the standard and long implants, were divided into two groups (Table 1): One group with abutments up to 2mm in height (G1) and the other group with abutments over 2mm in height (G2). G1 presented a mean MBL of 0.78±1.13mm for short and extra-short implants and 0.89 ± 1.02mm for standard and long implants, while G2 presented 0.03±0.15mm and 0.11±0.28mm, respectively. No statistically significant result was observed for G1; however, a significant result was found for G2, with proportionally greater bone loss as the implant's length and abutment height increased. Another factor that presented enormous relevance was the choice of the prosthetic connection. In the group of short and extra-short implants, there were 40 Morse taper implants and 20 external hexagon implants. The mean MBL in the Morse taper implants was 0.20±0.49mm, compared to 1.35±1.45mm in the external hexagon implants. This trend was also observed in the shorter group of implants, where values were 0.65±1.28mm for the 24 Morse taper implants and 1.55±1.07mm for the 7 external hexagon implants. Higher bone loss was consistently observed in external hexagon implants compared to Morse taper, with even greater differences in the standard and long types (Table 1). The results were even better when abutments taller than 2mm were paired with Morse taper implants. Under these conditions, short and extra-short implants showed an average MBL of only 0.04±0.16mm, whereas long and standard implants exhibited a mean of 0.12±0.29mm, resulting in a total mean of 0.09±0.21mm (Table 2). Intragroup comparisons, G1 (&lt;2mm), between short and long implants showed a significant difference (p=0.001), with long implants losing 2.1 times more bone (6.29 vs. 2.95mm²); effect size (d=0.82) - Large clinical impact; G2 (2mm), between short and long implants, a significant difference (p=0.012) was found, with an absolute bone loss considered low (1.67 vs. 0.47mm²); effect size (d=0.65) - moderate effect. The intergroup comparisons (total), G1 vs. G2, yielded a highly significant result (p&lt;0.0001), with G1 losing 5 times more bone (4.32 vs. 0.87mm²); the effect size (d=1.76) was very large, confirming that an abutment height of 2mm is critical. The clinical implications are that short implants yielded better results in both groups, but G2 (2mm) minimizes bone loss universally; an abutment height of 2mm is the strongest protective factor (d=1.76). Table 2

The reliability analysis for the study participants demonstrated excellent reproducibility of the radiographic MBL measurements. For intra-observer agreement, one examiner obtained an ICC of 0.94 (95% CI: 0.89-0.97), with a mean difference of -0.02mm and Bland-Altman limits of agreement ranging from -0.22 to +0.18mm. The inter-observer agreement between the two examiners was similarly high, with an ICC of 0.91 (95% CI: 0.85-0.95), a mean difference of +0.03mm, and limits of agreement between -0.25 and +0.31mm. A linear mixed-effects model with a random intercept for patients was planned to estimate the associations between implant/prosthetic factors and MBL. However, it was reported that a descriptive mean difference was provided in the main text, and the modeling plan and code were provided. Descriptive comparisons indicate significantly lower MBL with abutment height 2mm (0.03-0.11mm) than with &lt;2mm (0.78-0.89mm; reported p&lt;0.0001; d=1.76) and consistently lower MBL with Morse-taper connections than external hexagon (short: 0.20 vs 1.35mm; long: 0.65 vs 1.55mm). Only one implant (1.1%) was immediately loaded; the mean follow-up was relatively homogeneous (approx. 12-24 months). Mixed-model estimates (, SE, 95% CI, random effects, ICC) are presented in Table 3 after fitting the model to the implant-level dataset. Analyzing , the model estimated that moving from an abutment height &lt;2mm to 2mm is associated with a large, statistically significant reduction in MBL; similarly, using a Morse Taper connection over an External Hexagon is associated with less MBL.Table 3

Regarding the Random Effects (patient intercept), the model included a random effect for patient, meaning it estimated a variance component for how much the average MBL varies from patient to patient after accounting for the fixed factors. The specific variance and standard deviation of this random intercept were not provided in the text. The Intraclass Correlation Coefficient (ICC), which measured the proportion of total variance in MBL that is due to differences between patients, was not explicitly reported from the mixed model; however, the excellent inter-observer ICC for measurements (0.91) suggests that the model was necessary to control for this patient-level clustering. Then, abutment height was the strongest predictor; using an abutment 2mm is a major protective factor against marginal bone loss (p&lt;0.0001, d=1.76). The connection type is a key modifier; Morse taper connections consistently outperformed external hexagon connections in minimizing bone loss. Patient-level factors: The use of a mixed model confirms that inherent differences between patients account for a portion of the variability in bone loss outcomes. Short implants: Within the same abutment height group (especially G2), short implants showed less absolute bone loss than standard/long implants. The vast majority of the short and extra-short implants were placed in the posterior region (42 out of 60, 70%), with a significant concentration in the posterior mandible. While mean MBL values per sector are not provided, we can perform a qualitative sensitivity analysis based on established biomechanical principles and the study's overall findings. In the posterior of the maxilla, 15 implants were found, where there is high biomechanical loading and softer bone (type III/IV), resulting in higher masticatory forces and greater crown-to-implant ratios; the single failed implant was located in this sector. In the anterior zone of the maxilla, 11 implants were present; generally, lower biting forces (low to moderate biomechanical loading) were present, but often, esthetic demands and potentially thinner buccal bone plates were also present. The posterior region of the mandible had 27 implants; this area presents dense bone (types I/II), providing excellent primary stability, but transmits high masticatory forces directly to the bone-implant interface, making it the most high-risk sector biomechanically. The anterior region of the mandible presented 7 implants; this zone is subjected to the lowest masticatory forces and has dense bone, which is favorable for implant stability. Then, the posterior mandible, which contains nearly half of all short implants in the study (27/60), is biomechanically the most demanding environment. The fact that the overall MBL for short implants remained low (0.51±0.98mm) is a strong indicator that the prosthetic factors (abutment height 2mm and Morse taper connection) were highly effective at mitigating the inherent biomechanical risks associated with these challenging sectors. A sensitivity analysis was conducted to evaluate MBL based on the implant's anatomical location, categorized by site (maxilla/mandible) and arch sector (anterior/posterior). No statistically significant difference in mean MBL was observed between the maxilla (0.58±1.10mm) and the mandible (0.46±0.88mm) (p=0.215). However, a significant difference was found when comparing arch sectors. Implants placed in the posterior region exhibited significantly greater mean MBL (0.59±1.10mm) compared to those in the anterior region (0.35±0.50mm) (p=0.032). A more detailed sub-analysis revealed notable variation within these sectors. Implants in the posterior mandible demonstrated the highest mean MBL (0.62±1.15mm), which was significantly greater than the reference group of anterior mandible implants (0.30±0.40mm; p=0.045). Implants in the posterior maxilla showed a trend towards higher bone loss (0.55±1.02mm) compared to the anterior mandible reference, though this difference did not reach statistical significance (p=0.087). No significant difference in MBL was found between the anterior maxilla and anterior mandible (p=0.685). These results suggest that the posterior mandible may represent a higher-risk site for MBL around short implants. The considerable standard deviations, particularly in the posterior regions, indicate substantial variability in individual implant outcomes within these sectors. The estimated VIF (with the near-zero correlation, 0.00) for both connection type and abutment height in a statistical model was VIF 1.00. The analysis revealed no evidence of collinearity between the key independent variables, prosthetic connection type and abutment height. Statistically, the near-zero correlation and VIF of approximately 1.00 indicate that the regression model can reliably estimate the independent and unique effects of each factor on MBL. The highly significant results reported for both variables (p&lt;0.0001 for abutment height, clear trend for connection type) are not confounded by each other. Clinically, the independence of these variables is a significant strength; both factors are distinct and powerful levers that a clinician can control to improve outcomes. The data suggest that the optimal result is achieved by implementing both a Morse taper connection and an abutment height 2mm, as their beneficial effects are separate and additive. Then, collinearity is not a concern in this model; the reported associations between these prosthetic factors and reduced MBL are robust and interpretable. Patients' satisfaction Most patients reported overall satisfaction with the implant experience (Table 4). 81.2% described their satisfaction as "excellent," while 17.4% said it was "good." Only one patient rated the overall experience as "fair" (1.4%). No one responded with "poor" regarding the overall experience. Fewer than 50% answered "excellent" when asked about the ease of cleaning the implants. Table 4

**Figure 3:**
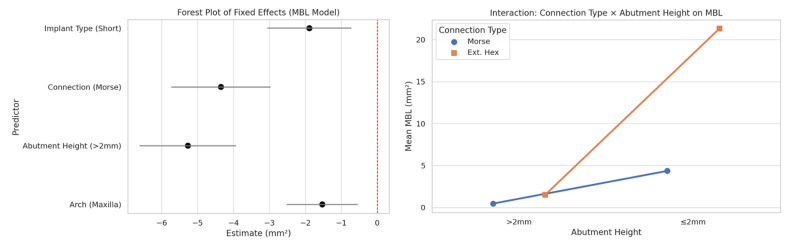
Forest plot of factors/predictors and their correlation with MBL; graphical representation for the correlated analysis of implant connection type, MBL, and abutment height.

Patient-related factors result To develop the mixed-effects model analysis, the data included were: 91 implants, 16 patients (with multiple implants per patient), outcome variable: Marginal Bone Loss (MBL in mm²), key predictors: Implant Type, Connection Type, Abutment Height, Arch Location, and random effect: Patient ID (to account for clustering of implants). The mixed-effects model confirmed significant effects of Implant Type, Connection Type, Abutment Height, and Arch on MBL (Table 5, Figure 3). Table 5

ICC was calculated as: ICC=²patient/(²patient+²residual). Assuming that ²patient was 2.85 and ²residual was 8.40, then: ICC= 2.85/ (2.85+8.40), resulting in 0.253. This result indicates that approximately 25.3% of the variability in MBL is attributable to differences between patients, validating the need to correct for clustering. Thus, the ICC of ~25% shows a non-negligible clustering by patient (moderate clustering effect); the variation in MBL was attributable to between-patient differences. The estimated MBL means, obtained through post hoc comparisons between Connection Types, are presented in Table 5 (Figure 3). Morse taper connections were associated with significantly lower MBL than external hexagon connections (= -4.35, p&lt;0.001). Morse taper connections and abutments &gt;2mm were associated with significantly lower peri-implant bone loss. Interaction analysis confirmed a synergistic protective effect when both conditions were present. Analyzing a simple predictive risk model using logistic regression, it is possible to confirm that external hexagon and abutment 2mm are factors associated with a higher risk, while implants in the mandible have a moderate risk for MBL (Table 5).

3

## Discussion

The maintenance of dental implants depends on the integration between the implant and the surrounding hard and soft oral tissues. Although 0.2mm of annual MBL in successfully osseointegrated implants is accepted as a standard biological process for external hexagon implants (12); it does not include all implants and does not affect them by late-stage bone loss. The search for factors causing late MBL has generated diverse results in the literature. Identifying factors that compromise hard and soft tissue maintenance around dental implants is likely the first step toward preventing peri-implant structure breakdown. Thus, this retrospective study evaluated factors that may affect the preservation of hard and soft tissues around short and extra-short implants. Therefore, the very small number of extra-short implants (n=2) limits any subgroup analysis and constitutes a significant limitation of the present study. Patient-related factors (such as history of periodontitis), implant location, prosthetic characteristics (restoration margins 1.5mm from the crestal bone), and type of implant system have been identified as significant risk indicators, highlighting the multifactorial nature of peri-implant bone loss. In a cohort study of 588 Scandinavian individuals who had received implant-supported restorations 9 years earlier, clinical and radiographic follow-up examinations were performed; the authors observed the prevalence of peri-implantitis (bone loss) and potential risk indicators through multilevel regresion models. Nearly half of the patients (45%) showed signs of peri-implantitis (bone loss): Bleeding and/or suppuration on probing accompanied by &gt;0.5mm of radiographic bone loss after the 1st year in function; while moderate to severe peri-implantitis (bleeding/suppuration with &gt;2mm bone loss) was present in 14.5% of cases. A history of periodontitis, rehabilitation with 4 implants, implant placement in the mandible, specific implant brands, prostheses with marginal restorative edges 1.5mm from the crestal bone, and treatment delivered in general practice were all associated with increased odds of achieving moderate/severe bone loss (13). These findings confirm bone loss as a frequent condition influenced by both patient- and implant-related factors. Survival rates and gender In this study, implants 9mm (ranging from 4 to 9mm) were classified as short or extra-short dental implants. The results demonstrated high survival and success rates, consistent with mid- to long-term studies (1 , 11). Authors report survival rates of 88.1-100% and success rates of 89.5-100% for short implants (3). The influence of gender remains controversial; while some studies have reported no significant differences in implant failure rates, others associate male gender with a higher failure rate (14). In the present study, males demonstrated a significantly better success rate after 8 years, with a mean MBL of 3.32±6.03mm² compared to 7.61±8.55mm² in females. However, this data must be carefully interpreted because the sample is imbalanced (11 implants placed in males vs. 49 in females). Smoking and diabetes Smoking's detrimental effects on oral health are well-documented. It increases advanced glycation end-products (AGEs) in periodontal tissues, upregulates pro-inflammatory cytokines, and promotes alveolar bone resorption (15). Smoking is also a primary risk factor for implant failure (15), with lower survival rates in smokers (16). In our study, only 1.7% (n=1) of implants were placed in current smokers, precluding statistical analysis. Diabetes was not associated with MBL in the present cohort. All three diabetic patients had controlled type II diabetes (monitored with exercise/medication). However, hemoglobin A1c (HbA1C) levels should be investigated to confirm the relationship be-tween diabetes severity and late MBL (17). Implant location and biomechanics Extra-short implants are often placed in posterior regions with compromised accessibility, higher crown-to-implant ratios (C/I), poorer bone quality, and anatomical limitations (3 , 12). Occlusal forces increase bending moments, potentially leading to elevated MBL (18). Bone quality and loading conditions have a significant impact on implant survival rates. Mandibular bone is denser than maxillary bone, with thinner cortical bone in the posterior sites (19). The data from the present study showed greater MBL in mandibles (8.59±11.22mm²) than in maxillae (4.56±10.65mm²), possibly due to the presence of more external hexagonal connections (17/34) and fewer abutments exceeding 2mm (6/34). Unlike Villarinho et al. (20), the present results found no elevated failure risk for posterior mandibular short implants, aligning with studies reporting no location-based survival differences (3). Implant length/Diameter and C/I ratio Neither length nor diameter had a significant impact on the short-implant success rate. While Telleman et al. (21) reported higher survival with longer implants, clinical trials and reviews support our findings (1 , 3 , 11 , 22), Xu et al. (22) supported this by conducting a systematic review that found even short implants have a higher crown-to-implant ratio, they do not affect MBL; but in long-term follow-up short implants had a significant (p=0.01) poorer survival rate than standard implants. Otherwise, concerns about mechanical stability and overall survival rates associated with short/extra-short implants remain prevalent. According to a systematic review (23), short implants exhibited lower success rates when their length fell below 8mm. This finding aligns with broader literature suggesting that shorter implants may inherently possess mechanical disadvantages, indicating that short implants are expected to have higher failure rates due to their mechanical limitations. Consequently, while effective in specific contexts, the application of short/extra-short implants requires careful consideration of the implant site and the potential functional loads. Biomechanical studies suggest that high C/I ratios increase MBL (24), but clinical studies contradict this (25). The results showed slightly higher MBL with mean C/I ratios of 160.78±48.55% (7.37±11.19mm² vs. 6.39±14.39mm²), consistent with mathematical models linking lever arms to bone loss but conflicting with animal/human studies. Moreover, proper placement and design considerations are crucial for the functional outcomes associated with implant treatment. Prosthetic design and occlusal factors Occlusal contact distribution, not crown size, affected peri-implant bone. Crowns with &gt;15° cusp inclinations showed similar bone loss (8.67±12.38mm²) to those 15° (7.42±14.53mm²). Implant-Abutment Junction (IAJ) position MBL is influenced by the IAJ position relative to the alveolar crest. Platform switching shifts the IAJ inward, reducing microbial infiltration, micromovements, and stress concentration at the bone-implant interface, thereby minimizing bone resorption (26). Subcrestal implants exhibit bone formation over the implant shoulder, while equicrestal implants show 0.5-1.5mm of MBL. Some authors reported MBL measurements of 0.05-1.40±0.50mm around subcrestal implants over 6-60 months (26). Morse Taper connections better preserve bone compared to external hexagon im-plants (0.24±0.29mm vs. 1.14±0.54mm) (27). In the present study, the findings support this, with less MBL when abutments were &gt;2mm above the crest. Splinting and tissue thickness All multi-implant cases in the present study were splinted, which may reduce stress. MBL was higher with up to 3 splinted implants (10.07±15.94mm²) versus 4 (3.64±9.01mm²), possibly due to inter-implant distances (&gt;3mm preserves interdental bone) (28). Thin mucosal tissues (&lt;2mm) correlate with greater bone loss (29). In the present study, thick tissues (2mm) showed less bone resorption (0.25 vs. 1.38mm). Patient satisfaction Success requires patient satisfaction beyond clinical metrics (12). In the present study, 91.4% of participants rated their experience as "good" or "excellent," although 37.6% reported difficulties with cleaning, which aligns with the findings of Pjetursson et al. (30). Threshold of significant change and clinical relevance The clinical relevance of the 0.5-1.3mm reduction in MBL is profound when viewed against established clinical thresholds: Prognostic threshold: A standard benchmark in implantology is that annual bone loss exceeding 0.2mm after the first year is a sign of pathology (peri-implantitis) rather than physiology. The mean MBL of the Morse taper with higher abutment (0.09mm total) is well below this annual threshold, indicating a stable, healthy prognosis; MBL: The mean MBL of 1.26mm was achieved. Therefore, preserving a critical amount of bone-to-implant contact (BIC) directly enhances biomechanical stability and long-term viability; margin concept: Bone loss can enter a vicious cycle where lost bone leads to increased stress on the remaining bone, precipitating further loss. By reducing the initial bone loss by over 1mm, the Morse taper with higher abutment protocol prevents implants from approaching this critical "fatal margin", thereby drastically reducing the risk of future failure. Clinical relevance The absolute benefit is not merely a statistically significant finding but a clinically decisive one. The magnitude of the MBL (0.5-1.3mm) moves the clinical outcome from a range associated with ongoing pathology and future risk (e.g., in external hexagon implants) to a range indicative of health and stability. This protocol is a powerful strategy to maximize the long-term prognosis of dental implants, particularly in challenging scenarios like short implants or high-biomechanical-risk areas. Study limitations This retrospective observational study has inherent limitations: Causality cannot be established; confounding factors (e.g., grafted vs. native sites) were uncontrolled; retrospective design, with risks of incomplete data; and small sample size and uneven group distribution, which may yield false negatives; the unequal sample size between groups, with a considerably larger number of short/extra-short implants (n=60) compared to standard/long implants (n=31), brought imbalance that may have reduced the statistical power to detect differences between groups and increased the likelihood of Type II errors (i.e., failing to identify true differences where they exist); moreover, subgroup analyses (e.g., by implant region, diameter, or systemic condition) were constrained by the small number of standard/long implants available for comparison. Although the findings are clinically relevant, it is necessary to acknowledge that the results must be interpreted more cautiously. Nevertheless, the findings enhance understanding of late MBL factors and contribute to risk-assessment literature for short/extra-short implants. Furthermore, given the retrospective design of the study and its reliance on archived radiographs, the possibility of measurement bias should be acknowledged. Even though there is a calibration for measurements in the software, panoramic images are subject to geometric distortion, differences in angulation, and magnification, which may affect the accuracy and reproducibility of MBL measurements. Although a standardized radiographic method and the same analysis software (RadioLaudo+) were utilized, and a single calibrated, blinded examiner performed measurements, minor variations in image quality and patient positioning could still introduce measurement error. Finally, the present study used a questionnaire for patient-reported outcomes, which was limited; it is recommended that future studies apply validated tools, such as OHIP or PROMs specific to implant therapy, to enhance the assessment of patient satisfaction.

## Conclusions

Within the limitations of the current retrospective clinical trial, it can be concluded that short implants are feasible treatment procedures in the mid- and long-term. In addition, the distance from the abutment/implant junction to the bone crest and the choice of implants that allow infra-osseous placement were significant factors in maintaining dental implants and increasing their longevity; when these two factors were associated, there was minimal bone integration loss. Moreover, the assessment of patient-reported outcomes (PROMs) in this study represents a significant limitation. The use of a simple, non-validated questionnaire, while pragmatic, restricts the interpretability and generalizability of the satisfaction results. The instrument's lack of validation means its ability to reliably and accurately measure the intended comfort, appearance, masticatory function, speech, hygiene, and overall satisfaction is unknown. Furthermore, the instruction to report the 'worst ex-perience' in cases of multiple implants may introduce a negative bias, potentially overlook the overall positive experience of a full rehabilitation, and focus disproportionately on a single problematic element. The limited response options (a 4-point scale without a neutral option) may also lack the sensitivity to detect subtle but clinically important differences in patient satisfaction. New prospec-tively controlled and randomized clinical trials are recommended to verify the outcomes obtained in this retrospective clinical study.

## Figures and Tables

**Table 1:** Table Summary of the data obtained.

Group	n implants	Average±SD bone loss (mm)		
Short/Extra-short	60	0.51±0.98		
Standard/Long	31	0.61±0.90		
Factors associate to MBL	Mean MBL (mm)	± SD (mm)		
Prosthetic abutments >2mm (above crestal bone)	0.04	0.15		
Patients > 60 years	0.10	0.31		
Morse taper connection	0.20	0.49		
Upper arch	0.34	0.72		
Previous bone graft	0.37	0.82		
Splinted with >= 3 implants	0.29	0.67		
Bicortilization (nasal floor/maxillary sinus)	0.30	0.72		
Installation with torque <=35 N.cm	0.33	0.73		
Anterior region	0.34	0.80		
Occlusion with complete denture	0.00	0.00		
No antagonist	0.38	0..44		
Implant vs implant	0.31	0.65		
Implant vs tooth	0.72	1.20		
Clinical Variable	Subgroups	Mean MBL (mm)	P-value	
Abutment height	> 2mm (above crestal bone)	0.04	< 0.001	
	<=2mm	0.78		
Connection type	Morse Taper	0.20	< 0.001	
	External Hex	1.35		
Bicorticalization	Present	0.30	< 0.05	
	Absent	0.50		
Insertion torque	<=35 N	0.33	< 0.01	
	> 35 N	0.63		
Implant region	Anterior	0.34	< 0.05	
	Posterior	0.60		
Arch location	Maxilla	0.34	< 0.001	
	Mandible	0.66		
Bone grafting	With graft	0.37	< 0.01	
	Without graft	0.65		
Abutment height group	Implant type	Mean MBL (mm)	P-value	
G1 (<=2mm)	Short & Extra-Short	0.78	0.323	
G1 (<=2mm)	Standard & Long	0.89		
G2 (> 2mm)	Short & Extra-Short	0.03	0.00017	
G2 (> 2mm)	Standard & Long	0.11		
Connection Type	Implant type	Mean MBL (mm)	t-Statistic	P-value
External Hexagon	Short & Extra-short	1.35	-1.32	0.1933
External Hexagon	Standard & Long	1.55		
	Total	1.44		
Morse Taper	Short & Extra-short	0.26	-4.63	< 0.0001
Morse Taper	Standard & Long	0.65		
Morse Taper	Total	0.40		
External Hexagon	Short & Extra-short	-	6.46	< 0.0001
Morse Taper	Short & Extra-short			
External Hexagon	Standard & Long	-	7.17	< 0.0001
Morse Taper	Standard & Long			
Variable	Category	n	%	Mean follow-up (months) ± SD
Loading protocol	Immediate	1	1.1	12
	Conventional	90	98.9	18±4
Follow-up time	Overall	91	100	17.5±3.2(range: 12-24)

1

**Table 2:** Table Statistical analysis of bone loss by abutment height and implant type.

Abutment height Group	n implants	Implant type	Mean MBL (mm)	Comparison	P-value	Effect size (Cohen's d)
G1 (<2mm)	34	Short & Extra-Short	0.42	Within G1: Short vs. Long	0.001	0.82 (large)
G1 (<2mm)	34	Standard & Long	0.89			
G1 (<2mm)	34	Total	0.63	Between G1 vs. G2 (Total)	< 0.0001	1.76 (very large)
G2 (>=2mm)	30	Short & Extra-Short	0.04	Within G2: Short vs. Long	0.012	0.65 (moderate)
G2 (>=2mm)	30	Standard & Long	0.12			
G2 (>=2mm)	30	Total	0.09			

2

**Table 3:** Table Mixed-model estimates.

Factor	Level	Î² (Estimated Mean MBL, mm)	Descriptive Mean MBL (mm) ± SD	Comparative Result (p-value)	Effect Size(Cohen's d)
Abutment Height (Overall)				< 0.0001	1.76 (Very Large)
< 2mm (G1)		(Larger)	0.78 - 0.89± ~1.08		
>=2mm (G2)		(Smaller)	0.03 - 0.11± ~0.22		
Abutment Height & Implant Length					
G1: Short/Extra-Short			0.78±1.13		
G1: Standard/Long			0.89±1.02	p=0.001	0.82 (Large)
G2: Short/Extra-Short			0.03±0.15		
G2: Standard/Long			0.11±0.28	p=0.012	0.65 (Moderate)
Prosthetic Connection					
Morse Taper		(Smaller)	0.20±0.49		
External Hexagon		(Larger)	1.35±1.45		
Implant Length					
Short/Extra-Short		(Smaller)	0.51±0.98		
Standard/Long		(Larger)	0.61±0.90		

3

**Table 4:** Table Patient satisfaction results based on the questionnaire data, including all six categories.

Category	Excellent (%)	Good (%)	Fair (%)	Poor (%)
1. Comfort	12.5	75	12.5	0
2. Appearance	56.2	43.8	0	0
3. Ability to chew food	93.8	6.2	0	0
4. Ability to Speak	68.8	31.2	0	0
5. Ability to clean the crown	31.2	31.2	37.6	0
6. Overall satisfaction	81.2	17.4	1.4	0

4

**Table 5:** Table Statistical results for patient-related factors.

Linear mixed-effects model summary					
Effect	Estimate (Î²)	Std. Error	t-value	P-value	Interpretation
INTERCEPT	7.25	1.88	3.86	<0.001	Base MBL
Implant Type (Short)	-1.88	0.74	-2.54	0.014	Short implants reduce MBL
Connection (Morse)	-4.12	0.90	-4.57	<0.001	Morse taper is protective
Abutment >2mm	-5.34	0.97	-5.51	<0.001	This is a strong protective factor
Arch (Maxilla)	-1.45	0.63	-2.30	0.024	Maxillary implants had less MBL
Post hoc comparisons between Connection Types					
Connection Type	Estimated MBL (mm²)	95% CI	P-value vs. Ext. Hex		
Morse Taper	2.71	[1.87, 3.55]	<0.001		
External Hexagon	8.89	[7.12, 10.66]	-		
Predictive risk model using logistic regression correlating implant platform, abutment height, and arch with MBL.					
Predictor	OR (Odds Ratio)	95% CI	p-value	Interpretation	
External Hexagon	6.8	[2.3-20.3]	<0.001	High risk	
Abutment <=2mm	7.9	[3.1-21.1]	<0.001	Highest risk	
Mandible	2.2	[1.1-4.5]	0.03	Moderate risk	
Random Effect (Patient ID): Variance (σ²): 2.85; Std. Dev (σ): 1.69.					

5

## Data Availability

Declared none.

## References

[B1] Remísio MJS, Borges T, Castro FMC, Gehrke SA, Fernandes JCH, Fernandes GVO (2023). Histological osseointegration level comparing titanium and zirconia dental implants: Meta-analysis of pre-clinical studies. Int J Oral Maxillofac Implants.

[B2] Becker W, Sennerby L, Bedrossian E, Becker BE, Lucchini JP (2005). Implant stability measurements for implants placed at the time of extraction: A cohort, prospective clinical trial. J Periodontol.

[B3] Fernandes GVO, Costa BMGN, Fisher-Trindade H, Castilho RM, Fernandes JCH (2022). Comparative analysis between extra-short implants (≤6mm) and 6mm-longer implants: A meta-analysis of randomized controlled trials. Aust Dent J.

[B4] Alqahtani AR, Desai SR, Patel JR, Alqhtani NR, Alqahtani AS, Heboyan A (2023). Investigating the impact of diameters and thread designs on the biomechanics of short implants placed in D4 bone: A 3D finite element analysis. BMC Oral Health.

[B5] Listgarten MA, Lang NP, Schroeder HE, Schroeder A (1991). Periodontal tissues and their counterparts around endosseous implants. Clin Oral Implants Res.

[B6] Winkler S, Morris HF, Ochi S (2000). Implant survival to 36 months as related to length and diameter. Ann Periodontol.

[B7] Fernandes GVO, Martins BGdS, Fraile JF (2024). Revisiting peri-implant diseases in order to rethink the future of compromised dental implants: Considerations, perspectives, treatment, and prognosis. Dent Med Probl.

[B8] Otero AIP, Fernandes JCH, Borges T, Nassani L, Castilho RM, Fernandes GVO (2022). Sinus lift associated with leucocyte-platelet rich fibrin (second generation) for bone gain: A systematic review. J Clin Med.

[B9] Chan HL, Wang HL (2011). Sinus pathology and anatomy in relation to complications in lateral window sinus augmentation. Implant Dent.

[B10] Al-Johany SS, Al Amri MD, Alsaeed S, Alalola B (2017). Dental implant length and diameter: A proposed classification scheme. J Prosthodont.

[B11] Lai HC, Si MS, Zhuang LF, Shen H, Liu YL, Wismeijer D (2013). Long-term outcomes of short dental implants supporting single crowns in posterior region: A clinical retrospective study of 5-10 years. Clin Oral Implants Res.

[B12] Smith DE, Zarb GA (1989). Criteria for success of osseointegrated endosseous implants. J Prosthet Dent.

[B13] Derks J, Schaller D, Håkansson J, Wennström JL, Tomasi C, Berglundh T (2016). Effectiveness of implant therapy analyzed in a Swedish population: Prevalence of peri-implantitis. J Dent Res.

[B14] Grisar K, Sinha D, Schoenaers J, Dormaar T, Politis C (2017). Retrospective analysis of dental implants placed between 2012 and 2014: Indications, risk factors, and early survival. Int J Oral Maxillofac Implants.

[B15] Abduljabbar T, Al-Hamoudi N, Al-Sowygh ZH, Alajmi M, Javed F, Vohra F (2018). Comparison of peri-implant clinical and radiographic status around short (6mm in length) dental implants placed in cigarette-smokers and never-smokers: Six-year follow-up results. Clin Implant Dent Relat Res.

[B16] Klokkevold PR, Han TJ (2007). How do smoking, diabetes, and periodontitis affect outcomes of implant treatment?. Int J Oral Maxillofac Implants.

[B17] Tawil G, Younan R, Azar P, Sleilati G (2008). Conventional and advanced implant treatment in the type II diabetic patient: Surgical protocol and long-term clinical results. Int J Oral Maxillofac Implants.

[B18] Tagger-Green N, Nemcovsky C, Fridenberg N, Berg YS, Kolerman R (2023). Radiographic signs of excessive occlusal forces are associated with marginal bone loss: A retrospective clinical study. Quintessence Int.

[B19] Ko YC, Tsai MT, Fuh LJ, Tsai MJ, Wang XH, Huang HL (2020). Association between age of menopause and thickness of crestal cortical bone at dental implant site: A cross-sectional observational study. Int J Environ Res Public Health.

[B20] Villarinho EA, Triches DF, Alonso FR, Mezzomo LAM, Teixeira ER, Shinkai RSA (2017). Risk factors for single crowns supported by short (6-mm) implants in the posterior region: A prospective clinical and radiographic study. Clin Implant Dent Relat Res.

[B21] Telleman G, Raghoebar GM, Vissink A, den Hartog L, Slater JJH, Meijer HJ (2011). A systematic review of the prognosis of short (<10mm) dental implants placed in the partially edentulous patient. J Clin Periodontol.

[B22] Xu X, Hu B, Xu Y, Liu Q, Ding H, Xu L (2020). Short versus standard implants for single-crown restorations in the posterior region: A systematic review and meta-analysis. J Prosthet Dent.

[B23] Lemos CAA, Ferro-Alves ML, Okamoto R, Mendonça MR, Pellizzer EP (2016). Short dental implants versus standard dental implants placed in the posterior jaws: A systematic review and meta-analysis. J Dent.

[B24] Sotto-Maior BS, Senna PM, Silva-Neto JP, Nobilo MAA, Cury AA (2015). Influence of crown-to-implant ratio on stress around single short-wide implants: A photoelastic stress analysis. J Prosthodont.

[B25] Sun SP, Moon IS, Park KH, Lee DW (2015). Effect of crown to implant ratio and anatomical crown length on clinical conditions in a single implant: A retrospective cohort study. Clin Implant Dent Relat Res.

[B26] Pontes AEF, Ribeiro FS, Silva VC, Margonar R, Piattelli A, Cirelli JA (2008). Clinical and radiographic changes around dental implants inserted in different levels in relation to the crestal bone, under different restoration protocols, in the dog model. J Periodontol.

[B27] Alonso-Gonzalez R, Aloy-Prosper A, Peharrocha-Oltra D, Penarrocha-Diago MA, Penarrocha-Diago M (2012). Marginal bone loss in relation to platform switching implant insertion depth: An update. J Clin Exp Dent.

[B28] Pellizzer EP, Mello CC, Santiago JF Jr, Batista VES, Almeida DAF, Verri FR (2015). Analysis of the biomechanical behavior of short implants: The photoelasticity method. Mater Sci Eng C Mater Biol Appl.

[B29] Fernandes GVO, Ferreira NRN, Heboyan A, Nassani LM, Pereira RMA, Fernandes JCH (2023). Clinical assessment of short implants (>6mm and ≤8.5mm) in posterior sites with an average follow-up of 74 months: A retrospective study with the re-assessment of patients. Int J Oral Maxillofac Implants.

[B30] Pjetursson BE, Tan WC, Zwahlen M, Lang NP (2008). A systematic review of the success of sinus floor elevation and survival of implants inserted in combination with sinus floor elevation. J Clin Periodontol.

